# Supplementary material for: The effect of lipid-lowering therapies on the pro-inflammatory and anti-inflammatory properties of vascular endothelial cells
Source: PLoS One. 2023 Feb 8;18(2):e0280741. doi: 10.1371/journal.pone.0280741 (PMC9907854; doi:10.1371/journal.pone.0280741)
Supplement: S1 Table — (DOCX) [file pone.0280741.s001.docx]

**Supplementary Table 1.** The primers used in the Real-Time PCR.

| **Gene** | **Primer** | **Nucleotide sequence 5’-3’** |
| --- | --- | --- |
| *IL1β* | sense  antisense | CACCAAGCTTTTTTGCTGTGAGT  GCACGATGCACCTGTACGA |
| *IL-10* | sense  antisense | GGCCCGACTATCTCGACTTT  GGTTGGATGTTCGTCCTCCT |
| *IL12A* | sense  antisense | TAACCAAGAATGAGAGTTGCCTA  TAAGGCACAGGGCCATCATAAA |
| *EBI3* | sense  antisense | AGAGCACATCATCAAGCCCG  CCCTGACGCTTGTAACGGAT |
| *TGFβ* | sense  antisense | AAATTGAGGGCTTTCGCCTT  GAACCCGTTGATGTCCACTTG |
| *IL18* | sense  antisense | TGCAATGTTTTCCTATTGCCTGT CCTGCTATTTTGCAGTGAACACA |
| *IL23* | sense  antisense | AGTGGAAGTGGGCAGAGATTC  CAGCAGCAACAGCAGCATTAC |
| *IL37* | sense  antisense | AAGACCTACGCCATGGGACATC  AAGACCTACGCCATGGGACATC |
| **Housekeeping**  **gene** | |  |
| *EF1-α* | sense  antisense | CTG AAC CAT CCA GGC CAA AT  GCC GTG TGG CAA TCC AAT |
