# Supplementary material for: The effect of lipid-lowering therapies on the pro-inflammatory and anti-inflammatory properties of vascular endothelial cells
Source: PLoS One. 2023 Feb 8;18(2):e0280741. doi: 10.1371/journal.pone.0280741 (PMC9907854; doi:10.1371/journal.pone.0280741)
Supplement: Attachment — Submitted filename: Response to Reviwer 2.docx [file pone.0280741.s002.docx]

**Reviewer #2: PLOS One Review 07/12/22 - PONE-D-22-07586**

**1. This article presents cytokine gene expression data and IL-35 concentrations from HUVECs stimulated with 25-OH-cholesterol that were treated or not with statins and/or ezetimibe. The authors conclude that there is no advantage of adding ezetimibe to statins over statin monotherapy. The manuscript addresses an important topic of interest. However, based on the in vitro experiments presented in this manuscript, this conclusion does not seem to be justified. The fact that ezetimibe in combination with statins under the specific experimental conditions does not further decrease the gene expression of pro-inflammatory cytokines in HUVECs, does not proof that such combinations are without any benefits to other tissues and cells relevant for arteriosclerosis formation. Ezetimibe inhibits intestinal cholesterol absorption, which in vivo prevents the inflammatory and damaging effects of cholesterol on vascular endothelium. While this study demonstrates that the anti-inflammatory effects of ezetimibe are minimal and do not enhance the anti-inflammatory effects of statins when added in combination, no statements regarding clinical benefits can be drawn from this study. The conclusions therefore should be changed accordingly, and the limitations of this in vitro study should be emphasized in the Discussion section of this manuscript. Furthermore, the study from Oh et al. (reference 35) compares high-dose rosuvastatin vs low-dose rosuvastatin combined with ezetimibe. Therefore, no group differences might indicate an advantage of the combination therapy, since lower doses may reduce adverse treatment effects. The statement in lines 248 to 250 seems therefore incorrect.**

We thank the Reviewer for this valuable suggestion. We changed the conclusions and the limitations of this study were emphasized in the Discussion section of this manuscript. The statement in lines 248 to 250 has been removed.

We added the below information in the manuscript:

Discussion

The main limitation of our work is that it is not possible to directly translate our *in vitro* findings into the process of atherosclerosis taking place *in vivo*. Our research suggests that, under these specific experimental conditions, ezetimibe in combination with statins does not further decrease the gene expression of pro-inflammatory cytokines in endothelial cells stimulated by 25-OHC *in vitro*; however, this does not mean that such combinations are without any benefits to other tissues and cells relevant for arteriosclerosis formation *in vivo*. Ezetimibe inhibits the absorption of cholesterol from the small intestine, leading to a reduction in intestinal cholesterol transmission to the liver, which protects the on vascular endothelium from the inflammatory and damaging effects of cholesterol *in vivo* [43]. Although our study showed that the anti-inflammatory effects of ezetimibe were minimal and did not enhance the anti-inflammatory effects of statins when added in combination, it is not possible to draw firm statements regarding clinical benefits. Therefore, further studies are needed to compare statin monotherapy with statin-ezetimibe combined therapy *in vivo*. (lines 302-314).

**2. The Method description is unclear and inconsistent with the statements in the Abstract and Results section of the manuscript. For example, the duration of culture of HUVECs varies throughout the manuscript between 12 and 24 hours.**

We thank the Reviewer. We corrected the incorrect description.

Materials and methods

Cells treatment in the viability level and gene expression

Gene expression

Enzyme-linked immunosorbent assay (ELISA) for IL-35

mRNA and supernatants were isolated after 20 hours of drug incubation, which corresponds to 24 hours of the experiment (four hours of pre-incubation with 25-OHC + 20 hours of drug incubation).

Figure 1 A-F. Relative expression of genes (A-F) in HUVECs prestimulated with 25-OHC (10 μg/mL) for four hours, followed by removal of oxysterol and stimulation for another 20 hours (i.e. 24-hours in total) with rosuvastatin (10 μM; 4815 ng/mL), atorvastatin (5 μM; 2793 ng/mL), ezetimibe (500 ng/mL), ezetimibe-rosuvastatin and ezetimibe-atorvastatin. Each experimental group has a control group that was not pre-stimulated with 25-OHC. Mean ± SD calculated from four biological replicates. Significant differences from negative controls are indicated by *P < 0.05; **P < 0.01; ***P < 0.001. Statistical analysis was conducted using one-way ANOVA and *post hoc* Tukey’s test.

Figure 2 A-C. Relative expression of gene *IL-35* (*EBI3*-A, *IL12*-B) and protein (C) in HUVECs prestimulated with 25-OHC (10 μg/mL) for four hours of incubation, followed by removal of oxysterol and stimulation for another 20 hours (i.e. 24-hour incubation in total) with rosuvastatin (10 μM; 4815 ng/mL), atorvastatin (5 μM; 2793 ng/mL), ezetimibe (500 ng/mL), ezetimibe plus rosuvastatin and ezetimibe plus atorvastatin. Each experimental group has a control group without pre-stimulation of 25-OHC. Mean ± SD calculated from four biological replicates. Significant differences from negative controls are indicated by *P < 0.05; **P < 0.01; ***P < 0.001. Statistical analysis was conducted using one-way ANOVA and *post hoc* Tukey’s test.

**3. Furthermore, the number of repetitions of the experiments remains unclear.**

We thank the Reviewer for this valuable suggestion. This sentence has been corrected.

We added the below information in the manuscript:

Statistical analysis

”Each analysis was performed in four independent experiments, with each experiment being repeated three times.” (lines 383-385).

**4. And the current manuscript version contains a number of grammar and spelling errors.**

English of the manuscript was carefully checked and corrected by an English native speaker (see the attached certificate).

**5. Abbreviations are not consistently defined upon their first use in the manuscript.**

We thank the Reviewer. Abbreviations were defined upon their first use in the manuscript.

**6. IL-35 is called anti-inflammatory in the abstract, but pro-inflammatory in the introduction and other parts of the manuscript.**

We thank the Reviewer for this valuable suggestion. This introductory paragraph was rephrased.

“A detailed analysis of the expression of pro-inflammatory *IL-1β, IL-18, IL-23,* and anti-inflammatory *IL35* (subunits *IL12A, EBI3*), transforming growth factor β (*TGF-β*)*, IL-10 and IL-37* was performed at the mRNA level. Additionally, IL-35 expression was determined at the protein level. “ (lines 62-66).

We added in the manuscript this information:

Discussion

”Previous studies indicate that IL-35 is a responsive anti-inflammatory cytokine, which is upregulated during inflammatory response, but not a constitutively expressed housekeeping cytokine. IL-35 is not constitutively expressed in tissues, being induced by pro-inflammatory stimuli, and inhibits lipopolysaccharide (LPS)-induced endothelial cell activation [27]. ” (lines 179-183).

”Furthermore, previous data suggest that IL-35 can suppress endothelial cell activation by inhibiting mtROS generation. Indeed, mtROS is known to contribute to inflammatory cytokine production and innate immune responses in macrophages and T cells [27]. (lines 211-214).

”Atorvastatin was found to inhibit dihydronicotinamide-adenine dinucleotide phosphate (NADPH)-oxidase dependent reactive oxygen species (ROS) generation; however, it showed no effect on mtROS generation, and activated IL-1β and IL-6 secretion in peripheral blood mononuclear cells (PBMC) from control and type 2 diabetes (T2D) patients. In turn, rosuvastatin mitigates coronary microembolization (CME)-induced cardiac injury by inhibiting NADPH-oxidase (Nox2)-induced ROS overproduction and alleviating apoptosis-related protein (p53/Bax/Bcl-2)-dependent cardiomyocyte apoptosis [35]. ’’ (lines 2515-221).

Complete the literature reference:

[27] Li X, Shao Y, Sha X, Fang P, Kuo YM, Andrews AJ, et al. IL-35 (interleukin-35) suppresses endothelial cell activation by inhibiting mitochondrial reactive oxygen species-mediated site-specific acetylation of H3K14 (histone 3 lysine 14). Arterioscler Thromb Vasc Biol. 2018;38: 599–609. doi:10.1161/ATVBAHA.117.310626

[35] Cao Y, Chen Z, Jia J, Chen A, Gao Y, Qian J, Ge J. Rosuvastatin Alleviates Coronary Microembolization-Induced Cardiac Injury by Suppressing Nox2-Induced ROS Overproduction and Myocardial Apoptosis. Cardiovasc Toxicol. 2022 Apr;22(4):341-351. doi: 10.1007/s12012-021-09716-4.

**7. Lines 77-79: “mRNA and supernatants were isolated after the 20 hours of drug stimulation. mRNA and supernatants were then isolated from HUVECs after 25-hydroxycholesterol (25-OHC) pre-stimulation and drug stimulation.” This seems to be duplicate statements.**

We thank the Reviewer, one sentence has been deleted.
~~mRNA and supernatants were isolated after the 20 hours of drug stimulation.~~

We added the below information in the manuscript:

Following 25-OHC pre-stimulation and drug stimulation, mRNA and the supernatants were isolated from HUVECs

**Line 94, similar lines 97-98: “…was added after 25-OHC prestimulation reduced the IL-18 mRNA level”. This seems to be a grammar error.**

We thank the Reviewer. This sentence has been corrected. Atorvastatin (p<0.001), rosuvastatin (p<0.05), and atorvastatin-ezetimibe (p<0.01) or rosuvastatin-ezetimibe (p<0.001) added after 25-OHC prestimulation also reduced *IL-18* mRNA levels.

**8. Line 138: “protine”. Please change to “protein”.**

We changed.

**9. Please add a reference for the statement in lines 154-156**

They also correspond to those in the plasma of patients receiving the highest therapeutic doses of these drugs: 80 mg for atorvastatin [13], 40 mg for rosuvastatin [14], and 10 mg for ezetimibe [15,16]. (lines 154-156).

13. Giordano A, Romano S, Monaco M, Sorrentino A, Corcione N, di Pace AL, et al. Differential effect of atorvastatin and tacrolimus on proliferation of vascular smooth muscle and endothelial cells. Am J Physiol - Hear Circ Physiol. 2012;302: 135–143. doi:10.1152/ajpheart.00490.2011

14.	Padró T, Lugano R, García-Arguinzonis M, Badimon L. LDL-induced impairment of human vascular smooth muscle cells repair function is reversed by HMG-CoA reductase inhibition. PLoS One. 2012;7. doi:10.1371/journal.pone.0038935

15.	Becher T, Schulze TJ, Schmitt M, Trinkmann F, El-Battrawy I, Akin I, et al. Ezetimibe inhibits platelet activation and uPAR expression on endothelial cells. Int J Cardiol. 2017;227: 858–862. doi:10.1016/j.ijcard.2016.09.122

16.	Kosoglou T, Statkevich P, Johnson-Levonas AO, Paolini JF, Bergman AJ, Alton KB. Ezetimibe: A review of its metabolism, pharmacokinetics and drug interactions. Clin Pharmacokinet. 2005;44: 467–494. doi:10.2165/00003088-200544050-00002

**10. Lines 162-164: “Further, the role of IL-37 may be related to the IL-18 pathway extracellularly and involved in the adhesion and transmigration of neutrophils in human coronary artery endothelial cells (HCAECs).” Please correct the sentence structure.**

We thank the Reviewer. This sentence has been corrected.

Furthermore, extracellular IL-37 may influence the IL-18 pathway, as well as the adhesion and transmigration of neutrophils in human coronary artery endothelial cells (HCAECs) [25].

**11.Lines 220: “for other analyzing genes”. Did you mean “for the other analyzed genes”?**

Yes, of course. We thank the Reviewer. This error has been corrected.

**12. Lines 239-240: ‘aniinflammatory effect”. Please correct to “anti-inflammatory effect”**

We changed.

**13. Line 241: What does the abbreviation “hsCRP” stand for?**

We thank the Reviewer. Abbreviations were defined upon their first use in the manuscript.

high-sensitivity C-reactive protein (hsCRP)

**14. Line 252: “Our study shows that 25-hydroxycholesterol can damage the endothelium …”. Cytokine gene expression changes alone do not proof any endothelial damage.**

We thank the Reviewer for this valuable suggestion. This sentence has been corrected. “It appears that 25-hydroxycholesterol can induce inflammation in endothelial cells.” (Pages , lines ).

**15. Line 263: “purchased in Lonza”. Please change to “purchased from Lonza”. Similar errors were repeated several times in the manuscript text.**

We thank the Reviewer for this valuable suggestion. We changed this.

**16 Line 265: “Rneasy Mini Kit”. Please change to “RNeasy Mini Kit”.** We changed.

**17. Line 270: “Cells treatment in the viability level and gene expression” This rephrase the title.**

### We thank the Reviewer for this valuable suggestion. We changed the title to: “Cell culture in monolayers” **18. Line 279: What is “proper medium”?**

We thank the Reviewer for this valuable suggestion. This sentence has been corrected.

We added the below information in the manuscript:

”Both trypsinized HUVECs were separately seeded on 24-well plates at a density of 100,000 cells per well in a 600 µl EGM-2.”

**19. Could the authors pleased explain why 25-hydroxycholesterol was removed prior to treatment with statins and/or ezetimibe, since in vivo high cholesterol levels would be present throughout the treatment with these drugs.**

We added the below information in the manuscript:

”High-fat meals (are rich with the triglycerides (TG) and oxysterols: 25-hydroxycholesterol, 7 a-hydroxycholesterol, 7 b-hydroxycholesterol, a-epoxycholesterol, b-epoxycholesterol and 7-ketocholesterol) can impair endothelial function within three to four hours, which is associated with peak postprandial lipemia [17]. In healthy people, plasma TG levels peak three to four hours after the ingestion of a high-fat meal, and tend to return to baseline within six to eight hours [18]. Therefore, 25-OHC stimulation was stopped after four hours. ’’ (lines 157-163)

Moreover, we used the same research model in our previous works: doi: 10.1016/j.biopha.2022.112679; doi: 10.3390/ijms21061953.10.1080/21688370.2021.1956284; doi: 10.1371/journal.pone.0256996.

**Line 292: “G Gene expression”. Did the authors mean “Gene expression”?**

Yes, of course. We thank the Reviewer. This error has been corrected.

**Line 301: Livak and Schmittgen 2001: This reference is not included in the reference list.**

We thank the Reviewer. This error has been corrected.

[49] Livak KJ, Schmittgen TD. Analysis of relative gene expression data using real-time quantitative PCR and the 2-ΔΔCT method. Methods. 2001;25: 402–408. doi:10.1006/meth.2001.1262
 **Line 317: “Shapiro-Will test”. Please correct to “Shapiro-Wilk test”.**

We thank the Reviewer. This error has been corrected.

**Lines 320-322: “The individual analysis was performed in nine-four independent experiments, while each experiment was repeated twice or three times depending on the method.” This description seems unclear.**

We thank the Reviewer for this valuable suggestion. This sentence has been corrected.

We added the below information in the manuscript:

Statistical analysis

” Each analysis was performed in four independent experiments, with each experiment being repeated three times.”
