# Supplementary material for: The effect of lipid-lowering therapies on the pro-inflammatory and anti-inflammatory properties of vascular endothelial cells
Source: PLoS One. 2023 Feb 8;18(2):e0280741. doi: 10.1371/journal.pone.0280741 (PMC9907854; doi:10.1371/journal.pone.0280741)
Supplement: Attachment — Submitted filename: PLoS ONE review 110622.docx [file pone.0280741.s003.docx]

Page 3: “*Statins, a class of cholesterol-lowering medications - low-density lipoprotein (LDL) that inhibit 3-hydroxy-3-methyl-glutaryl-coenzyme A reductase. This drugs are commonly administered to treat atherosclerotic cardiovascular disease (CVD).*” Please correct these sentences, which seem grammatically incorrect.

Page 4: “…*Analysis of the expression of genes pro-inflammatory cytokines”. Please change to “Analysis of the expression of genes of pro-inflammatory cytokines”.* Likewise for page 5:“…*Analysis of the expression of genes anti--inflammatory cytokines”*

Page 4: “*The administration of 25-hydroxycholesterol was associated with an increase in IL-18 mRNA (p<0.01).*” According to the corresponding figure, p should be < 0.001.

Page 7: “*The present study focuses on key inflammatory factors that play important role in the endothelium [19].”* Please change to “…*that play an important role*…”

Page 7: Please correct the spelling for “*preiovusly*”.

Page 8: “*Previous studies indicate that IL-35 is a responsive anti-inflammatory cytokine, which is upregulated during inflammatory response, but not a constitutively expressed housekeeping cytokine. IL-35 is not constitutively expressed in tissues, being induced by pro-inflammatory stimuli, and inhibits lipopolysaccharide (LPS)-induced endothelial cell activation [27].*”

Please change to “*Previous studies indicate that IL-35 is a responsive anti-inflammatory cytokine, which is upregulated during inflammatory responses, but not a constitutively expressed housekeeping cytokine. IL-35 is not constitutively expressed in tissues, but is induced by pro-inflammatory stimuli, and inhibits lipopolysaccharide (LPS)-induced endothelial cell activation [27]*.”

Page 8: “*In turn TGF-β, plays a key role in proper cell activity and is widely considered an anti-inflammatory cytokine.*”

Please change to: “*In turn TGF-β, which plays a key role in proper cell activity, is widely considered an anti-inflammatory cytokine.”*

Page 8: “*Atorvastatin and rosuvastatin, have*…”. Please remove the comma.

Page 8: Please be consistent in the tenses used. For example “*Zhang et al. (2018) [29] report that the administration of atorvastatin (5 mg/kg/day) was associated with a reduction of IL-1β in the serum of rabbits. Furthermore, Satoh et al. (2014) [30] presented…*”

Page 8: “…in human monocytic cells line derived from an acute monocytic leukemia patients…” Please change to “…in human monocytic cell lines derived from an acute monocytic leukemia patient…”

Page 8 to 9: Please correct the spelling of “*It has bene reported* …”

Page 9: “…*can reduce IL-23 serum level*” Please change to “…*can reduce IL-23 serum levels*”.

Page 9: “…*is in line with the Shin et al. (2017) [34].”* Please change to “…*is in line with Shin et al. (2017) [34].”*
