# Supplementary material for: The effect of lipid-lowering therapies on the pro-inflammatory and anti-inflammatory properties of vascular endothelial cells
Source: PLoS One. 2023 Feb 8;18(2):e0280741. doi: 10.1371/journal.pone.0280741 (PMC9907854; doi:10.1371/journal.pone.0280741)
Supplement: Attachment — Submitted filename: Answers to the Reviewer 2 comments.docx [file pone.0280741.s004.docx]

**The suggestions and questions raised by Reviewer #2**

Reviewer #2: The revised manuscript is significantly improved and all reviewer questions have been addressed. There are a couple of remaining regarding the last sentence of the abstract and one statement in the method section of the revised manuscript.

Abstract: “The anti-inflammatory effect of the combination therapies appears to be based on the effects of the statins alone and not their combination with ezetimibe; therefore, they offer no advantage over statin monotherapy.” The last part of this statement seems incorrect, since the fact that combination therapies with statins and ezetimibe do not provide enhanced anti-inflammation in HUVECs over statin monotherapy does not mean that they offer no advantage.

We thank the Reviewer for this valuable suggestion. The sentence has been removed from the abstract.

Page 14: “After reaching 80–90 % conﬂuence, HUVECs were stimulated with 25-hydroxycholesterol (10 µg/ml) for four hours. After incubation, the cells were centrifuged, the compound was discarded, and HUVECs were stimulated with atorvastatin (5 µM), rosuvastatin (10 µM) and ezetimibe (500 ng/ml) for 24 hours. After incubation, the cells were centrifuged, the compounds were discarded, and the cells were resuspended in EGM-2 medium.” This description appears confusing and the timing should probably read 20 hours instead of 24 hours.

We thank the Reviewer for this valuable suggestion. We changed the description:

After reaching 80–90 % conﬂuence, HUVECs were stimulated with 25-hydroxycholesterol (10 µg/ml) for four hours. After incubation, the cells were centrifuged, the compound was discarded, and HUVECs were stimulated with atorvastatin (5 µM), rosuvastatin (10 µM) and ezetimibe (500 ng/ml) for 20 hours. Total cell stimulation was 24 hours: four hours of pre-incubation with 25-OHC + 20 hours of drug incubation. After incubation, the cells were centrifuged, the compounds were discarded, and the cells were resuspended in EGM-2 medium.”

Since PLoS ONE does not provide proof reading, please revise the following spelling and grammar errors as outlines below:

Page 3: “Statins, a class of cholesterol-lowering medications - low-density lipoprotein (LDL) that inhibit 3-hydroxy-3-methyl-glutaryl-coenzyme A reductase. This drugs are commonly administered to treat atherosclerotic cardiovascular disease (CVD).” Please correct these sentences, which seem grammatically incorrect.

We corrected this sentence:

The statins are cholesterol-lowering medications that inhibit the activity of 3-hydroxy-3-methyl-glutaryl-coenzyme A reductase. These drugs are commonly administered to treat atherosclerotic cardiovascular disease (CVD).

Page 4: “…Analysis of the expression of genes pro-inflammatory cytokines”. Please change to “Analysis of the expression of genes of pro-inflammatory cytokines”. Likewise for page 5:“…Analysis of the expression of genes anti--inflammatory cytokines”.

We have corrected these sentences. Analysis of the expression of genes of pro-inflammatory cytokines and Analysis of the expression of genes anti--inflammatory cytokines

Page 4: “The administration of 25-hydroxycholesterol was associated with an increase in IL-18 mRNA (p<0.01).” According to the corresponding figure, p should be < 0.001.

We thank the Reviewer for this valuable suggestion, we have corrected the error.

Page 7: “The present study focuses on key inflammatory factors that play important role in the endothelium [19].” Please change to “…that play an important role…”

We thank the Reviewer for this valuable suggestion, we have corrected the error.

Page 7: Please correct the spelling for “previously”.

We thank the Reviewer for this valuable suggestion, we have corrected the error.

Page 8: “Previous studies indicate that IL-35 is a responsive anti-inflammatory cytokine, which is upregulated during inflammatory response, but not a constitutively expressed housekeeping cytokine. IL-35 is not constitutively expressed in tissues, being induced by pro-inflammatory stimuli, and inhibits lipopolysaccharide (LPS)-induced endothelial cell activation [27].”
Please change to “Previous studies indicate that IL-35 is a responsive anti-inflammatory cytokine, which is upregulated during inflammatory responses, but not a constitutively expressed housekeeping cytokine. IL-35 is not constitutively expressed in tissues, but is induced by pro-inflammatory stimuli, and inhibits lipopolysaccharide (LPS)-induced endothelial cell activation [27].”

We thank the Reviewer for this valuable suggestion, we have corrected the error.

Page 8: “In turn TGF-β, plays a key role in proper cell activity and is widely considered an anti-inflammatory cytokine.”
Please change to: “In turn TGF-β, which plays a key role in proper cell activity, is widely considered an anti-inflammatory cytokine.”

We thank the Reviewer for this valuable suggestion, we have corrected the error.

Page 8: “Atorvastatin and rosuvastatin, have…”. Please remove the comma.

We thank the Reviewer for this valuable suggestion, we have corrected the error.

Page 8: Please be consistent in the tenses used. For example “Zhang et al. (2018) [29] report that the administration of atorvastatin (5 mg/kg/day) was associated with a reduction of IL-1β in the serum of rabbits. Furthermore, Satoh et al. (2014) [30] presented…”

We thank the Reviewer for this valuable suggestion, we have corrected the error.

Page 8: “…in human monocytic cells line derived from an acute monocytic leukemia patients…” Please change to “…in human monocytic cell lines derived from an acute monocytic leukemia patient…”
We thank the Reviewer for this valuable suggestion, we have corrected the error.

Page 8 to 9: Please correct the spelling of “It has bene reported …”

We thank the Reviewer for this valuable suggestion, we have corrected the error.

Page 9: “…can reduce IL-23 serum level” Please change to “…can reduce IL-23 serum levels”.

We thank the Reviewer for this valuable suggestion, we have corrected the error.

Page 9: “…is in line with the Shin et al. (2017) [34].” Please change to “…is in line with Shin et al. (2017) [34].”

We thank the Reviewer for this valuable suggestion, we have corrected the error.

Page 10: Please correct the spelling of “tool-like receptor 2”.

We thank the Reviewer for this valuable suggestion, we have corrected the error.

Page 10: Please change “…IL-6 a in trastuzumab-treated mice” to “IL-6 in trastuzumab-treated mice”.

We thank the Reviewer for this valuable suggestion, we have corrected the error.

Page 12: Please correct the spelling of “Apopoliprotein E (ApoE)”.

We thank the Reviewer for this valuable suggestion, we have corrected the error.

Page 13: Please change “…which protects the on vascular endothelium…” to “…which protects the vascular endothelium…”.

We thank the Reviewer for this valuable suggestion, we have corrected the error.

Page 14: Is the following correct? “…human recombinant E.coli, lyophilized powder (R3-IGF-1)…”

The following statement is correct.

Page 14: Please change “…gentamicin (GA-1000), and heparin, fetal bovine serum (FBS), …” to “… gentamicin (GA-1000), heparin, and fetal bovine serum (FBS), …”.

We have corrected the error.

Page 14: Please change “Atorvastatin, rosuvastatin, ezetimibe, 25-hydroxycholesterol (25-OHC) and using primers were bought from Sigma-Aldrich (USA).” To “Atorvastatin, rosuvastatin, ezetimibe, 25-hydroxycholesterol (25-OHC) and primers were bought from Sigma-Aldrich (USA).”

We have corrected the error.

Please 14: Please change “RNeasy Mini Kit was bought in Qiagen (Germany).” To “RNeasy Mini Kit was bought from Qiagen (Germany).”

We have corrected the error.

Page 14: Please correct this sentence “Both trypsinized HUVECs were separately seeded on 24-well plates at a density of 100,000 cells per well in a 600 µlEGM-2.”

We have corrected the error.
Page 18 reference 13: Please correct the abbreviated name of the cited journal.

We have corrected the error.
